# Activity of cefepime/enmetazobactam against highly multidrug-resistant bacterial isolates recovered from war-associated wounds in Ukraine

**DOI:** 10.1093/jacamr/dlaf256

**Published:** 2026-01-21

**Authors:** Jale Boral, Nora Toft, Alp Eren Baybes, Chaitanya Tellapragada, Oleksandr Nazarchuk, Celine Fernandez, Christian Giske, Kristian Riesbeck

**Affiliations:** Clinical Microbiology, Department of Translational Medicine, Lund University, Malmö, Sweden; Clinical Microbiology, Department of Translational Medicine, Lund University, Malmö, Sweden; Clinical Microbiology, Department of Translational Medicine, Lund University, Malmö, Sweden; School of Medicine, Koç University, Istanbul, Türkiye; Division of Clinical Microbiology, Department of Laboratory Medicine, Karolinska Institute, Stockholm, Sweden; Center for Thermal Injury and Plastic Surgery, Vinnytsya Regional Clinical Hospital Named After Pirogov, Vinnytsya, Ukraine; Department of Microbiology, National Pirogov Memorial Medical University, Vinnytsya, Ukraine; Medical Department, ADVANZ Pharma, Helsingborg, Sweden; Division of Clinical Microbiology, Department of Laboratory Medicine, Karolinska Institute, Stockholm, Sweden; Department of Clinical Microbiology, Karolinska University Hospital, Stockholm, Sweden; Clinical Microbiology, Department of Translational Medicine, Lund University, Malmö, Sweden; Department of Clinical Microbiology, Skåne University Hospital, Lund, Sweden

## Abstract

**Background:**

Escalating resistance among Gram-negative pathogens limits β-lactam options. Cefepime/enmetazobactam combines a fourth-generation cephalosporin with a class A β-lactamase inhibitor. We evaluated its activity against multidrug, extensive and pandrug-resistant war-wound isolates from Ukraine.

**Materials and methods:**

We tested 215 clinical samples (2022–2023) isolated from wounded patients in Ukraine by broth microdilution. Paired comparisons of cefepime monotherapy versus cefepime/enmetazobactam was done to evaluate enmetazobactam. Whole-genome sequencing (*n* = 83) identified β-lactamases, sequence types and outer-membrane protein alterations.

**Results:**

Across Enterobacterales, resistance decreased from 60.5% (26/43) with cefepime to 27.9% (12/43) with the cefepime/enmetazobactam combination. In *K. pneumoniae*, resistance declined from 92.6% (75/81) to 74% (60/81) and in *P. mirabilis*, from 88.9% (8/9) to 11.1% (1/9), respectively. *P. aeruginosa* showed a modest change [77.8% (23/36) to 58.3% (32/36)], while *A. baumannii* remained non-susceptible to both cefepime and cefepime/enmetazobactam with the rates of 91.3% (42/46) to 82.6% (36/46), respectively. Genotype–phenotype correlation revealed that in *K. pneumoniae*, reduced activity was associated with OmpK36 loop-3 insertions, OmpK35 loss and metallo-β-lactamases, whereas selected sequence types lacking these changes were more susceptible. In *A. baumannii*, OXA-type carbapenemases and *bla*ADC beta-lactamases predominated; isolates lacking type VI secretion, *omp33-36* and adhesin genes (*ata/bap*) demonstrated relatively higher susceptibility to cefepime/enmetazobactam.

**Conclusions:**

Cefepime/enmetazobactam improved susceptibility relative to cefepime, most notably in Enterobacterales, but activity was constrained by permeability defects and non-target β-lactamases. Combination therapy with agents that does not get hydrolysed by MBLs and integrated β-lactamase detection with practical permeability indicators may optimize the clinical use of this combination in high-resistance settings.

## Introduction

Emerging antimicrobial resistance among Gram-negative pathogens has progressively diminished the efficacy of β-lactams, motivating the development of β-lactam/β-lactamase inhibitor combinations. Cefepime/enmetazobactam pairs the fourth-generation cephalosporin cefepime with the penam-sulfone inhibitor enmetazobactam. The combination drug received regulatory approval for the treatment of complicated urinary tract infection after the phase III ALLIUM trial showed higher microbiological eradication at day 14 compared with piperacillin/tazobactam.^1^ Cefepime displays broad activity and relative stability to many class C and some class D β-lactamases, but its efficacy is reduced by ESBLs, porin loss, efflux upregulation or PBP alterations.^2^ Enmetazobactam enhances cefepime’s activity by inhibiting class A β-lactamases including TEM, SHV, CTX-M and inhibitor-resistant variants.^3^

Surveillance data show that adding enmetazobactam restores cefepime potency against Enterobacterales.^4^ PK/PD studies further support target attainment in ESBL-producing *Klebsiella pneumoniae*.^5^ However, activity remains limited as the combination is minimally active against MRSA, *Enterococcus* spp. and anaerobes, consistent with cephalosporin-based inhibitor combinations and product labelling.^6^ Critically, metallo-β-lactamases (MBLs; e.g. NDM, VIM) are not inhibited by enmetazobactam; activity against KPC producers is reduced relative to carbapenem–boronate or diazabicyclooctane beta-lactam/beta-lactamase inhibitor comparators, and the combination shows no added value over cefepime alone against *P. aeruginosa* and *A. baumannii* in recent multi-centre testing of carbapenem-resistant Gram negatives.^4,7^ Although activity against OXA-type carbapenemases (e.g. OXA-23, OXA-24/40, OXA-58, OXA-48-like) is minimal, occasional partial susceptibility has been reported in OXA-48-like Enterobacterales lacking major permeability defects or co-produced MBLs.^7^ In comparison with carbapenem, cefepime shows partial structural resilience to hydrolysis by certain OXA-48-like enzymes that hydrolyse cephalosporins less efficiently.^8^

Non-enzymatic mechanisms including decreased outer-membrane permeability, porin loss, efflux pump overexpression and PBP alterations can elevate MICs regardless of carbapenemases, highlighting the need for susceptibility-guided use.^9,10^ These mechanisms may limit activity of novel combinations such as aztreonam-avibactam that addresses the inhibition of MBLs, ESBLs and OXA-like carbapenemases.^11^ Similar to aztreonam-avibactam, outer-membrane porins (OMPs) are central determinants of cefepime/enmetazobactam activity in Enterobacterales. β-lactams traverse the outer membrane through trimeric porins; in *K. pneumoniae*, OmpK35 (OmpF-like) and OmpK36 (OmpC-like) are the principal channels, and loss-of-function, down-regulation or constricting loop mutations (e.g. L3 alterations) reduce β-lactam influx and raise MICs, particularly when combined with ESBL or AmpC enzymes.^9^ Such permeability defects blunt the pharmacodynamic benefit of β-lactamase inhibition, whereas preserved porins correlate with lower MICs^6,9^ In *P. aeruginosa*, intrinsic low permeability with MexAB-OprM efflux limits β-lactam entry which explains the limited added value of enmetazobactam.^4,12^ In *A. baumannii*, alterations in OM proteins such as CarO and combined permeability–efflux mechanisms contribute to poor activity of cefepime/enmetazobactam.^6^

In this study, we evaluated cefepime/enmetazobactam against highly drug-resistant Enterobacterales, *P. aeruginosa*, *A. baumannii* and *P. mirabilis* isolates originating from conflict zones in Ukraine.^13–15^

## Materials and methods

### Susceptibility testing

A total of 215 war zone wound isolates that were obtained from Ukraine (2022–2023) were tested for cefepime and cefepime/enmetazobactam susceptibility using broth microdilution method according to the European Committee on Antimicrobial Susceptibility Testing (EUCAST) guidelines.

### Whole-genome sequencing and bioinformatic analysis

Eighty-three out of 215 isolates were sequenced as published in the previous studies of the same research group.^14,15^ Forty-six of these isolates were *A. baumannii* and 37 of them were *K. pneumoniae*. Briefly, Pasteur Scheme was used to conduct multi-locus sequence typing. Antimicrobial resistance genes and virulence determinants for *A. baumannii* isolates were identified using ABRicate (Version 1.0.0.) and virulence finder database (VFDB) while Kleborate (https://github.com/klebgenomics/Kleborate) was used for the identification of resistance genes and virulence profiling of *K. pneumoniae* isolates.

### Available sequence data

All sequences have been submitted to the National Center for Biotechnology Information (https://www.ncbi.nlm.nih.gov); SUB15232350 and BioProject ID PRJNA1246223 for *A. baumannii* isolates and SUB14387023 and BioProject ID PRJNA1102281 for *K. pneumoniae* isolates.

### Statistical analysis

Paired categorical comparisons of cefepime versus cefepime/enmetazobactam were performed using McNemar’s exact test (two-sided) on susceptible/resistant strains defined by clinical MIC breakpoints (4 mg/L for all species except *P. aeruginosa*, 8 mg/L). Analyses were done using GraphPad Software v.10 (Prism, La Jolla, CA, USA).

## Results

### Comparative susceptibility to cefepime/enmetazobactam versus cefepime monotherapy

**Figure 1.**
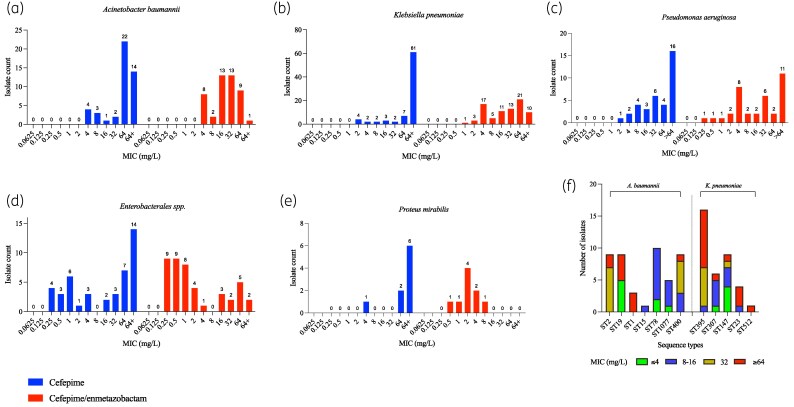
MIC distribution of isolates for cefepime and cefepime/enmetazobactam. (a) *Acinetobacter baumannii* (*n* = 46), (b) *Klebsiella pneumoniae* (*n* = 81), (c) *Pseudomonas aerginosa* (*n* = 37), (d) Enterobacterales (*n* = 42), (e) *Proteus mirabilis* (*n* = 8) and (f) Frequency of various sequence types categorized on the basis of MIC values.

Across all species, cefepime/enmetazobactam exhibited lower MIC50 values than cefepime alone, indicating greater *in-vitro* potency (Table 1). An evident shift to lower MICs was observed in Enterobacterales followed by minor effects in *P. aeruginosa* and *A. baumannii* (Figure 1). Among Enterobacterales, cefepime MIC50/MIC90 was 32/64 mg/L versus 1/64 mg/L for the combination. In *K. pneumoniae*, the MIC50 decreased from 64 to 32 mg/L (MIC90 remained 64 mg/L). *Proteus mirabilis* showed a prominent shift from 64/64 to 2/4 mg/L. By contrast, *P. aeruginosa* demonstrated modest changes (cefepime 32/64 mg/L; combination 32/64 mg/L), and *A. baumannii* showed limited improvement (cefepime 64/64 mg/L; combination 16/64 mg/L). In Enterobacterales, resistance decreased from 60.5% to 27.9% (*P* < 0.0001). In *K. pneumoniae*, resistance decreased from 92.6% to 74.1% (*P* = 0.0003). In *P. mirabilis*, resistance went down from 88.9% to 11.1% (*P* = 0.0156) while in *P. aeruginosa*, a decrease from 77.8% to 58.3% (*P* = 0.0391) was observed. *A. baumannii* remained highly resistant (91.3% to 82.6%, *P* = 0.2891).

**Table 1.** MIC50 and MIC90 of all isolates to cefepime and cefepime/enmetazobactam with resistance rates and statistical evaluation

Pathogen	Antibiotic	Range	MIC50 (mg/L)	MIC90 (mg/L)	Resistance (%)	*P* value (NcNemar exact test)
*A. baumannii* (*n* = 46)	Cefepime	≤4 to ≥64	64.0	64.0	91.3	0.2891
	Cefepime/ Enmetazobactam	≤4 to ≥64	16.0	64.0	82.6	0.2891
Other Enterobacterales (*n* = 43)	Cefepime	≤0.25 to ≥64	32.0	64.0	60.5	<0.0001
	Cefepime/ Enmetazobactam	≤0.25 to ≥64	1.0	64.0	27.9	<0.0001
*K. pneumoniae* (*n* = 81)	Cefepime	≤2 to ≥64	64.0	64.0	92.6	0.0003
	Cefepime/ Enmetazobactam	≤1 to ≥64	32.0	64.0	74.1	0.0003
*P. aeruginosa* (*n* = 36)	Cefepime	≤0.25 to ≥64	64.0	64.0	77.8	0.0391
	Cefepime/ Enmetazobactam	≤0.25 to ≥64	32.0	64.0	58.3	0.0391
*P. mirabilis* (*n* = 8)	Cefepime	≤4 to ≥64	64.0	64.0	88.9	0.0156
	Cefepime/ Enmetazobactam	≤0.5 to ≥8	2.0	4.0	11.1	0.0156

### Impact of beta-lactamase positivity, sequence types and outer-membrane protein alterations on susceptibility

Across 83 sequenced isolates (*K. pneumoniae n* = 37; *A. baumannii n* = 46), cefepime/enmetazobactam increased the proportion categorized as susceptible compared with cefepime alone (Table S1, available as Supplementary data at *JAC-AMR* Online). In *K. pneumoniae*, susceptibility increased from 0% with cefepime to 13.5% with the combination antibiotic of cefepime/enmetazobactam. It was observed that susceptibility varied by sequence type. ST147 demonstrated the highest susceptibility to the combination antibiotic (44%), while ST307 was intermediate (17%) and ST395, ST23 and ST512 isolates remained uniformly resistant (Figure 1f). Genotype to phenotype patterns were consistent with permeability effects. The isolates with *OmpK36* GD-loop alterations showed a lower susceptible proportion to the combination antibiotic (4.5%) than isolates without this trait (27%) (Table S1). Moreover, alteration of *OmpK35* was associated with non-susceptibility (6.2% versus 60.0% when *OmpK35* was intact). Although MBLs frequently coincided with resistance, even MBL-negative isolates were largely resistant, underscoring porin status as a major determinant.

In *A. baumannii*, ST19 showed the highest susceptibility (55.6%), whereas ST2, ST1 and ST400 were predominantly resistant. Beta-lactamase profiles aligned with these outcomes. Isolates carrying OXA-type carbapenemases together with *bla*ADC beta-lactamases were largely non-susceptible to both cefepime and the combination. Class A GES enzymes correlated with the highest MICs and an absence of isolates classified as susceptible to the combination antibiotic, while GES-negative subsets showed higher susceptibility rate (25%).

## Discussion

In our study, cefepime/enmetazobactam increased susceptibility relative to cefepime alone, with variation by species, sequence type, β-lactamase profile and permeability. In Enterobacterales, paired analyses showed a clear benefit in isolates dominated by class A ESBLs, aligning with enmetazobactam’s inhibitory spectrum and multi-centre surveillance data on restoration of cefepime activity against CTX-M, TEM, and SHV producers.^4^ In *P. mirabilis*, enmetazobactam showed greater success whereas *P. aeruginosa* showed only a minor change due to low permeability and resistance-nodulation-division efflux pumps.^4,16^

The 37 *K. pneumoniae* war isolates previously studied by our group were largely extensively drug resistant and pandrug resistant, explaining why the combination improved susceptibility yet left most isolates non-susceptible.^14^ The carbapenemase content was dominated by *bla*NDM, alone or with *bla*OXA-48-like, which is outside the inhibitory range of enmetazobactam.^11^ Our genotypic evaluation suggested that porin alterations reduced predicted drug entry. OmpK36 loop 3 insertions and OmpK35 defects correlated with lower susceptibility, consistent with experimental work that channel constriction and porin loss diminish β-lactam influx.^12,17^ Sequence types harbouring these defects (e.g. ST395) responded poorly whereas subsets of ST147 without these mutations responded better despite similar ESBL profiles, indicating that porin status can outweigh beta-lactamase content.^17^ The 46 *A. baumannii* isolates, corresponding to our published war-wound cohort showed low susceptibility to combination, which was expected given the highly drug-resistant profile, OXA-type carbapenemases with *bla*ADC, and outer-membrane adaptations.^15,18^ ST19 isolates lacking the type VI secretion system (T6SS), *omp33-36* and adhesin encoding genes *ata*/*bap* showed the highest susceptibility to cefepime/enmetazobactam, supporting permeability and adhesin status as additional determinants of resistance.^19^

Although the isolates originated from multiple centres in Ukraine, clonal relatedness should be taken into consideration, which potentially limits the variety of outcome as the variance in OMP defects and carbapenemase positivity is low, as noted previously.^13,15^ Another limitation of this study is not having the whole cohort of isolates whole-genome sequenced. Further studies should be done to assess the high success of cefepime/enmetazobactam in *P. mirabilis* isolates regarding their clonal relatedness, carbapenemase positivity and OMP profiles. Since no EUCAST/CLSI breakpoints or ECOFFs exist for cefepime/enmetazobactam in *A. baumannii*, we applied the cefepime PK/PD non-species related breakpoint (≤4 mg/L) as an exploratory threshold for comparative purposes. This approach allowed relative, but not definitive, categorization of susceptibility to highlight trends across species.

Our studies suggest that cefepime/enmetazobactam should be positioned as a preferred β-lactam option for ESBL-mediated Enterobacterales when outer-membrane permeability is preserved as it reliably outperforms cefepime alone as a carbapenem-sparing option, with special attention in multidrug-resistant infections caused by *P. mirabilis*. Despite the higher cost, it may be cost-effective in stewardship-focused settings aiming to reduce carbapenem use. Mechanism-guided selection integrating rapid β-lactamase detection with permeability markers will maximize the clinical value of cefepime/enmetazobactam and support antimicrobial stewardship.

## Supplementary Material

dlaf256_Supplementary_Data

## Supplementary data

Table S1 is available as Supplementary data at *JAC-AMR* Online.
