# Supplementary material for: Activity of cefepime/enmetazobactam against highly multidrug-resistant bacterial isolates recovered from war-associated wounds in Ukraine
Source: JAC Antimicrob Resist. 2026 Jan 21;8(1):dlaf256. doi: 10.1093/jacamr/dlaf256 (PMC12820527; doi:10.1093/jacamr/dlaf256)
Supplement: dlaf256_Supplementary_Data [file dlaf256_supplementary_data.docx]

**Table S1**. Comparative MICs of cefepime and cefepime/enmetazobactam, sequence types, beta-lactamase positivity, and OMP profiles of whole-genome sequenced isolates (*n*=83).

|  | **Isolate Code** | **Sequence Type** | **MIC cefepime/enmetazobactam (mg/L)** | **MIC cefepime (mg/L)** | **Class A (Serine β-lactamases)** | **Class B (Metallo-β-lactamases)** | **Class C (AmpC Cephalosporinases)** | **Class D (OXA-type β-lactamases)** | **OMP sequences** |
| --- | --- | --- | --- | --- | --- | --- | --- | --- | --- |
| ***Klebsiella pneumoniae*** | KR6069 | 395 | >64 | >64 | TEM-1, CTX-M-15, SHV-11 | NDM-1 |  | OXA-1, OXA-48 | truncated ompK35, altered ompK-36* |
|  | KR6131 | 395 | 32 | >64 | TEM-1, CTX-M-15, SHV-1 | NDM-1 |  | OXA-1, OXA-48 | truncated ompK35, altered ompK-36* |
|  | KR6135 | 395 | 64 | >64 | CTX-M-15, SHV-11 |  |  | OXA-1, OXA-48 | truncated ompK35, altered ompK-36* |
|  | KR6142 | 395 | 32 | >64 | TEM-1, CTX-M-15, SHV-11 | NDM-1 |  | OXA-1, OXA-48 | truncated ompK35, altered ompK-36* |
|  | KR6143 | 395 | 64 | >64 | TEM-1, CTX-M-15, SHV-11 | NDM-1 |  | OXA-1, OXA-48 | truncated ompK35, altered ompK-36* |
|  | KR6144 | 395 | 64 | >64 | TEM-1, CTX-M-15, SHV-11 | NDM-1 |  | OXA-1, OXA-48 | truncated ompK35, altered ompK-36* |
|  | KR6030 | 395 | >64 | 64 | TEM-1, CTX-M-15, SHV-11 | NDM-1 |  | OXA-1, OXA-48 | truncated ompK35, altered ompK-36* |
|  | KR6084 | 395 | 64 | >64 | TEM-1, CTX-M-15, SHV-11 |  |  | OXA-1, OXA-48 | truncated ompK35, altered ompK-36* |
|  | KR6058 | 395 | 32 | >64 | TEM-1, CTX-M-15, SHV-11 | NDM-1 |  | OXA-1, OXA-48 | truncated ompK35, altered ompK-36* |
|  | KR6063 | 395 | >64 | >64 | TEM-1, CTX-M-15, SHV-11 | NDM-1 |  | OXA-1, OXA-48 | truncated ompK35, altered ompK-36* |
|  | KR6066 | 395 | 32 | >64 | TEM-1, CTX-M-15, SHV-11 | NDM-1 |  | OXA-1 | truncated ompK35 |
|  | KR6067 | 395 | 16 | >64 | TEM-1, CTX-M-15, SHV-11 | NDM-1 |  | OXA-1 | truncated ompK35 |
|  | KR6077 | 395 | 64 | 64 | TEM-1, CTX-M-15, SHV-11 | NDM-1 |  | OXA-1, OXA-48 | truncated ompK35 |
|  | KR6080 | 395 | >64 | >64 | - | NDM-1 |  |  | truncated ompK35, altered ompK-36* |
|  | KR6081 | 395 | 32 | >64 | TEM-1, CTX-M-15, SHV-11 | NDM-1 |  | OXA-1 | truncated ompK35 |
|  | KR6149 | 395 | 64 | >64 | TEM-1, CTX-M-15, SHV-11 | NDM-1 |  | OXA-1, OXA-48 | truncated ompK35, altered ompK-36* |
|  | KR6163 | 395 | 32 | >64 | TEM-1, CTX-M-15, SHV-11 |  |  | OXA-48 | truncated ompK35, altered ompK-36* |
|  | KR6098 | 307 | 8 | >64 | TEM-1, CTX-M-15, SHV-28 | NDM-1 |  |  | truncated ompK35 |
|  | KR6108 | 307 | 16 | >64 | TEM-1, CTX-M-15, SHV-28 | NDM-1 |  |  | truncated ompK35 |
|  | KR6122 | 307 | 64 | >64 | TEM-1, CTX-M-15, SHV-28 | NDM-1 |  | OXA-1 | truncated ompK35 |
|  | KR6078 | 307 | 16 | >64 | TEM-1, CTX-M-15, SHV-28 | NDM-1 |  |  | truncated ompK35 |
|  | KR6079 | 307 | 4 | >64 | TEM-1, CTX-M-3, SHV-28 | NDM-1 |  |  | truncated ompK35 |
|  | KR6060 | 307 | 16 | >64 | TEM-1, CTX-M-15, SHV-28 | NDM-1 |  |  | truncated ompK35 |
|  | KR6145 | 147 | 4 | 32 | TEM-1, CTX-M-15, SHV-11 | NDM-1 |  | OXA-9 | - |
|  | KR6156 | 147 | 4 | >64 | CTX-M-15, SHV-11 | NDM-1 |  |  | - |
|  | KR6166 | 147 | 4 | 64 | TEM-1, CTX-M-15, SHV-11 | NDM-1 |  | OXA-1, OXA-9, OXA-48 | truncated ompK35, altered ompK-36* |
|  | KR6071 | 147 | 8 | >64 | CTX-M-15, SHV-11 | NDM-1 |  |  | - |
|  | KR6072 | 147 | 8 | >64 | TEM-1, SHV-11 |  |  | OXA-9, OXA-48 | truncated ompK35, altered ompK-36* |
|  | KR6162 | 147 | 32 | >64 | TEM-1, CTX-M-15, SHV-11 | NDM-1 |  | OXA-1, OXA-9, OXA-48 | truncated ompK35, altered ompK-36* |
|  | KR6007 | 147 | 64 | >64 | TEM-1, CTX-M-15, SHV-11 | NDM-1 |  | OXA-1, OXA-9, OXA-48 | truncated ompK35, altered ompK-36* |
|  | KR6029 | 147 | 4 | 32 | TEM-1, CTX-M-15, SHV-11 | NDM-1 |  | OXA-9 | - |
|  | KR6047 | 147 | 8 | >64 | CTX-M-15, SHV-11 | NDM-1 |  |  | - |
|  | KR6068 | 23 | >64 | >64 | CTX-M-15, SHV-1 | NDM-6 |  | OXA-1 | truncated ompK35, altered ompK-36* |
|  | KR6083 | 23 | 16 | >64 | CTX-M-15, SHV-1 |  |  | OXA-1 | truncated ompK35, altered ompK-36* |
|  | KR6085 | 23 | >64 | >64 | CTX-M-15, SHV-1 | NDM-6 |  | OXA-1 | truncated ompK35, altered ompK-36* |
|  | KR6159 | 23 | 64 | >64 | SHV-1 |  |  | OXA-1 | truncated ompK35, altered ompK-36* |
|  | KR6061 | 512 | 64 | >64 | KPC-3 |  |  |  | truncated ompK35, altered ompK-36* |
|  |  |  |  |  |  |  |  |  |  |
| ***Acinetobacter baumannii*** | KR6155 | 2 | 32 | 64 | PER-7 |  | ADC-30 | OXA-23, OXA-66 | - |
|  | KR6146 | 2 | 32 | 64 | PER-7 |  | ADC-30 | OXA-23, OXA-66 | - |
|  | KR6137 | 2 | 32 | >64 | PER-7 |  | ADC-30 | OXA-23, OXA-66 | - |
|  | KR6156 | 2 | 64 | 64 | PER-7 |  | ADC-30 | OXA-23, OXA-66 | - |
|  | KR6151 | 2 | 64 | 8 |  |  |  | OXA-23, OXA-66 | - |
|  | KR6158 | 2 | 32 | 64 | PER-1 |  | ADC-11 | OXA-72, OXA-66 | - |
|  | KR6148 | 2 | 32 | >64 | PER-1 |  | ADC-11 | OXA-72, OXA-66 | - |
|  | KR6113 | 2 | 32 | 64 | PER-1 |  | ADC-11 | OXA-72, OXA-66 | - |
|  | KR6124 | 2 | 32 | >64 | PER-1 |  | ADC-11 | OXA-72, OXA-66 | - |
|  | KR6133 | 19 | 4 | 4 |  |  | ADC267 | OXA-69 | loss of omp33-36 |
|  | KR6118 | 19 | 4 | 8 |  |  | ADC267 | OXA-69 | loss of omp33-36 |
|  | KR6140 | 19 | 4 | 32 |  |  | ADC267 | OXA-69 | loss of omp33-36 |
|  | KR6127 | 19 | 4 | 4 |  |  | ADC267 | OXA-69 | loss of omp33-36 |
|  | KR6132 | 19 | 4 | 8 |  |  | ADC267 | OXA-69 | loss of omp33-36 |
|  | KR6052 | 19 | 64 | >64 | GES-12 |  | ADC-185 | OXA-69, OXA-72 | loss of omp33-36 |
|  | KR6038 | 19 | 64 | >64 | GES-12 |  | ADC-185 | OXA-69, OXA-72 | loss of omp33-36 |
|  | KR6086 | 19 | 64 | >64 | GES-12 |  | ADC-185 | OXA-69, OXA-72 | loss of omp33-36 |
|  | KR6104 | 19 | 64 | >64 | GES-12 |  | ADC-185 | OXA-69, OXA-72 | loss of omp33-36 |
|  | KR6053 | 1 | 64 | 64 | GES-11 |  | ADC-240 | OXA-69, OXA-23 | loss of omp33-36 |
|  | KR6051 | NA | 64 | >64 | GES-11 |  | ADC, ADC-240 | OXA-69, OXA-23 | - |
|  | KR6112 | 1 | 64 | 64 |  |  | ADC-240 | OXA-69, OXA-23 | - |
|  | KR6147 | 15 | 16 | 4 |  |  |  | OXA-72 | - |
|  | KR6109 | 78 | 16 | 64 | CARB-16 |  | ADC-152 | OXA-72, OXA-51 | - |
|  | KR6121 | 78 | 16 | 64 | CARB-16 |  | ADC-152 | OXA-72, OXA-51 | - |
|  | KR6089 | 78 | 16 | >64 | CARB-16 |  | ADC-152 | OXA-72, OXA-51 | - |
|  | KR6136 | 78 | 4 | >64 | CARB-16 |  | ADC-152 | OXA-72, OXA-51 | - |
|  | KR6150 | NA | 16 | 16 |  |  | ADC-152 | OXA-72, OXA-51 | - |
|  | KR6070 | 78 | 16 | 32 | CARB-16 |  | ADC-152 | OXA-72, OXA-51 | - |
|  | KR6031 | 78 | 16 | >64 | CARB-16 |  | ADC-152 | OXA-72, OXA-51 | - |
|  | KR6033 | 78 | 8 | 64 | CARB-16 |  | ADC-152 | OXA-72, OXA-51 | - |
|  | KR6045 | 78 | 8 | >64 |  |  | ADC-152 | OXA-72, OXA-51 | - |
|  | KR6028 | 78 | 4 | 64 | CARB-16 |  | ADC-152 | OXA-72, OXA-90 | - |
|  | KR6111 | 1077 | 16 | 64 |  |  | ADC-152 | OXA-72, OXA-90 | - |
|  | KR6153 | 1077 | 16 | 64 | CARB-16 |  | ADC-152 | OXA-72, OXA-90 | - |
|  | KR6026 | 1077 | 4 | 64 | CARB-16 |  | ADC-152 | OXA-72, OXA-90 | - |
|  | KR6110 | 1077 | 16 | >64 | CARB-16 |  | ADC-152 | OXA-72, OXA-90 | - |
|  | KR6154 | 1077 | 16 | 4 | CARB-16 |  | ADC-152 | OXA-72, OXA-90 | - |
|  | KR6161 | 400 | 32 | 64 | GES-11 |  | ADC | OXA-100 | loss of omp33-36 |
|  | KR6160 | 400 | 32 | 64 | GES-11 |  | ADC | OXA-100 | loss of omp33-36 |
|  | KR6114 | 400 | 32 | 64 | GES-11 |  | ADC | OXA-100 | loss of omp33-36 |
|  | KR6128 | 400 | 16 | 64 | GES-11 |  | ADC | OXA-100 | loss of omp33-36 |
|  | KR6141 | 400 | 32 | 64 | GES-11 |  | ADC | OXA-100 | loss of omp33-36 |
|  | KR6139 | 400 | 16 | 64 | GES-11 |  | ADC | OXA-100 | loss of omp33-36 |
|  | KR6120 | 400 | 16 | 64 | GES-11 |  | ADC | OXA-100 | loss of omp33-36 |
|  | KR6117 | 400 | 32 | 64 | GES-11 |  | ADC | OXA-100 | loss of omp33-36 |
|  | KR6062 | 400 | >64 | >64 |  |  | ADC | OXA-100 | loss of omp33-36 |

*Altered in OmpK36 corresponds to isolates with GD ( Glycine-Aspartate) insertions in the L3 loop.
